# Fat Oxidation during Exercise in People with Spinal Cord Injury, and Protocols Used: A Systematic Review

**DOI:** 10.3390/healthcare10122402

**Published:** 2022-11-29

**Authors:** Soraya Martín-Manjarrés, Javier Leal-Martín, Cristina Granados, Esmeralda Mata, Ángel Gil-Agudo, Irene Rodríguez-Gómez, Ignacio Ara

**Affiliations:** 1 Hospital Nacional de Parapléjicos, SESCAM, 45004 Toledo, Spain; 2 GENUD-Toledo Research Group, Universidad de Castilla-La Mancha, 45071 Toledo, Spain; 3 CIBER on Frailty and Healthy Aging, Instituto de Salud Carlos III (CIBERFES, ISCIII), 28029 Madrid, Spain; 4 Departamento de Educación Física y Deporte, Facultad de Educación y Deporte, Universidad del País Vasco (UPV/EHU), 01007 Vitoria, Spain; 5 Facultad Ciencias del Deporte, Universidad de Castilla-La Mancha, 45071 Toledo, Spain; 6 Departamento de Medicina Física y Rehabilitación, Hospital Nacional de Parapléjicos, SESCAM, 45004 Toledo, Spain

**Keywords:** Fat_max_, exercise metabolism, paraplegia, energy metabolism, physical activity

## Abstract

Background: The aim of this study was to summarize evidence on energy metabolism through peak fat oxidation (PFO) and maximum fat oxidation (Fat_max_), as well as to analyze the protocols used in people with spinal cord injury (SCI) and to examine the main factors related to fat oxidation ability (i.e., age, sex, level of physical activity, and level and degree of injury). Methods: Studies to determine PFO and Fat_max_ using indirect calorimetry with an arm exercise protocol for SCI patients were included after a systematic search. Other endpoints included study design, sample size, control group, demographic data, level of injury, physical condition, protocol, outcomes measured, and statistical findings. Results: Eight studies (n = 560) were included. The mean value of VO_2peak_ was 1.86 L∙min^−1^ (range 0.75–2.60 L∙min^−1^) (lowest value in the tetraplegic subjects). The PFO ranged between 0.06 and 0.30 g∙min^−1^ (lowest rates: the non-trained subjects with cervical SCI; highest: the tetraplegic subjects). Two types of exercise protocol were found: arm cycle ergometer, and wheelchair propulsion with a computerized ergometer. Five studies used an incremental protocol (2–3 min/stage, different load increments); the rest performed tests of 20 min/stage at three intensities. Conclusion: There are few existing studies measuring fat oxidation in SCI, many of which used small and heterogeneous samples. PFO was lower in SCI subjects when compared with non-injured people performing lower-limb exercise; however, comparing upper-limb exercise, people with SCI showed higher values.

## 1. Introduction

Spinal cord injury (SCI) is associated with increased prevalence of metabolic [1,2,3,4] and cardiovascular diseases [5] compared to the general population. Some of the contributing factors could include the decrease in muscle mass together with the increase in the percentage of body fat (%BF) [6,7], the sedentary lifestyle, and the decrease in physical activity. The role of muscular denervation is also important [8], as are the changes in glycogenolysis and gluconeogenesis [9].

As shown in a classic study by Bauman et al., 1994 [10], a higher prevalence of diabetes mellitus (22% vs. 6%) and glucose intolerance (34% vs. 12%) is observed in subjects with SCI compared to subjects without SCI. These alterations seem to be related to a high level of injury, increased percentage of body fat (%BF), and the male sex [11]. Moreover, cardiovascular disease is more frequent and has an earlier onset in people with SCI compared to the general population [12]. In fact, cardiovascular disease has become the leading cause of death in people with SCI, causing 46% of deaths in people who have lived more than 30 years with an SCI, as well as 35% of deaths in those over 60 years old, regardless of the duration of the SCI [13]. Likewise, people with SCI may present changes in their response and tolerance to physical exercise as a consequence of the aforementioned alterations, but also due to the loss of sensory and motor function and the effects on the autonomic nervous system [14].

The energy required during exercise is provided by fat and carbohydrates, and there is a shift in the mobilization and utilization of these substrates as intensity increases [15]. The fat rate increases from low to moderate intensity and then decreases when exercise becomes more intense [16]. If the fat oxidation rates are depicted versus the intensity of the exercise, expressed as a percentage of peak oxygen consumption (%VO_2peak_), this will increase up to a certain point and then decrease, showing peak fat oxidation (PFO). The relative intensity at which PFO is reached is called Fat_max_. Furthermore, other factors—such as sex, physical condition, or nutritional status—influence Fat_max_ during exercise [17]. PFO and Fat_max_ have been largely associated with insulin sensitivity, metabolic flexibility, and reductions in metabolic risk factors [18,19]. For this reason, analyzing PFO and Fat_max_ during physical exercise can be an important predictor of cardiometabolic health in both healthy populations and those with various pathologies. To this end, exercise protocols have been developed to reliably determine PFO and Fat_max_, providing reference values for specific populations [20]. However, there are no reviews about fat oxidation during exercise—or the protocols used—in this population with SCI, although such a review could be relevant for the prevention of cardiovascular disease and metabolic syndrome. Likewise, given that SCIs cause different consequences in terms of disconnection in the central nervous system depending on the involvement and level of the injury [21], consideration of this factor is also essential.

Therefore, the primary objective of this paper was to review the existing studies on energy metabolism in people with SCI that measure PFO and Fat_max_, and to analyze the protocols that have been used. The secondary objective was to examine the main characteristics of subjects with SCI (i.e., age, sex, level of physical activity, and level and degree of injury) that are related to their ability to oxidize fat during physical exercise.

## 2. Materials and Methods

This review follows the recommendations of the PRISMA statement [22].

### 2.1. Study Selection

**Figure 1:**
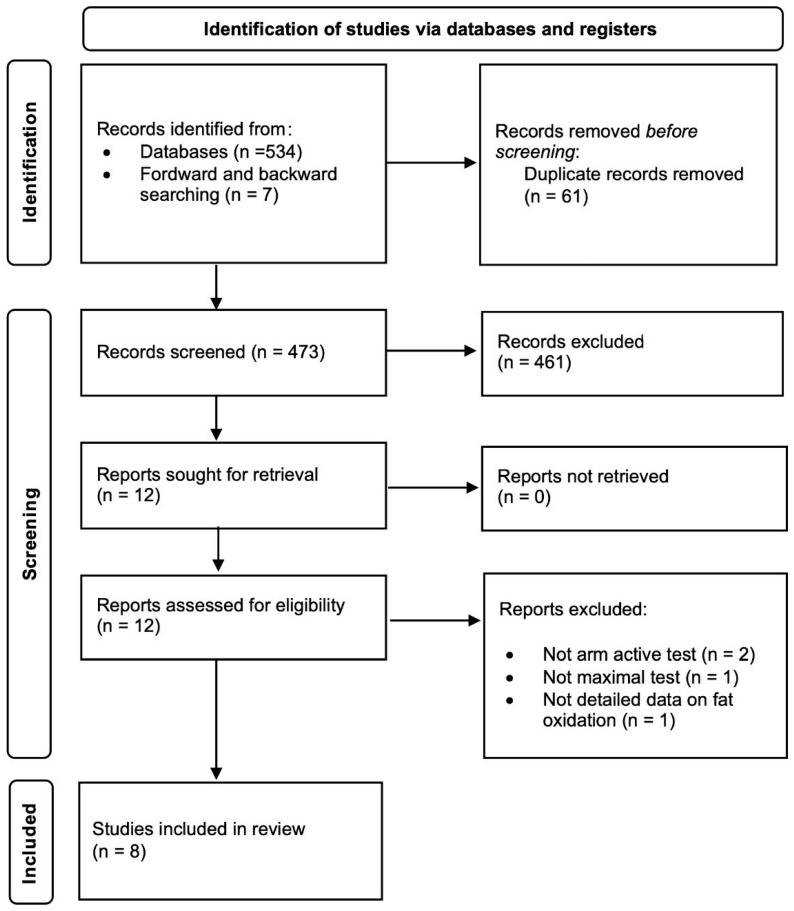
Flow diagram.

A PICOS (participants, intervention, comparators, study outcomes, and study design) approach was used to rate studies for eligibility [23]. Therefore, the search included studies carried out in people with SCI who were older than 18 years and performed an arm exercise protocol at different intensities to determine PFO and Fat_max_ based on gas analysis through indirect calorimetry. Observational and intervention studies published in English and Spanish were included. The exclusion criteria were studies on wheelchair users that did not specify that at least 75% of the included participants had an SCI. Two independent reviewers screened the titles and abstracts retrieved by the search and excluded the studies that were not eligible. Twelve studies were retrieved in full text and, after reading them, eight were selected to be included in the review (Figure 1).

### 2.2. Search Strategy

The search was carried out in PubMed and in the Cochrane Trial Database. In addition, a reverse manual search was carried out on the references of the articles included in the review. The date of the last search carried out was 2 February 2022. The search was limited to articles published since 2002—the year in which the Fat_max_ protocols were developed [24]. Details of the search strategy used are included in Figure 1.

### 2.3. Data Extraction

Two independent reviewers abstracted data from the full-text articles using a sample data extraction form. The collected data included study design, sample size, presence or absence of control group, demographic data (e.g., sex, age), level of injury, physical condition (e.g., sedentary, trained, athlete), protocol used for the fat oxidation test, outcomes measured (VO_2peak_, PFO, Fat_max_), and findings (statistical differences). The author of one of the studies [25] was contacted by e-mail to request data not provided in the published document.

### 2.4. Quality Assessment

The quality of the selected articles (Table 1) was evaluated using the STROBE scale [26] for the 6 observational studies, which obtained a score equal to or greater than 17/22. The two randomized clinical trials scored more than 7/10 on the PEDro scale [27].

## 3. Results

From the 560 studies identified, after removing duplicates and screening according to inclusion and exclusion criteria, 8 studies were included in the present review. Table 2 presents a synthesis of the main data collected in them.

### 3.1. Design of Studies

Six of the included studies had observational cross-sectional designs [28,29,30,31,32,33], while two were clinical trials [33,34]. Four of the observational studies had a control group [29,31,32,33]. One of the clinical trials had a crossover design [34], while the other was a parallel-group study [35].

### 3.2. Sample Characteristics

The sample sizes were between 8 and 21 participants, not counting the control groups (CGs). The total population of all of the studies was 91 subjects of both sexes (84 men and 7 women) and >18 years old (range: 22 to 64 years). Of the total of 91 participants, 85 subjects with SCI were included—in some studies exclusively [28,33,34,35], and in others as a majority alongside other pathologies such as poliomyelitis (n = 1) [30], spina bifida (n = 3) [29,30], or limb amputation (n = 3) [29,31]. Four studies [29,31,32,33] included non-injured subjects as a control group (n = 45; 44 men, 1 woman).

Most of the studies classified their SCI participants according to the American Spinal Injury Association (ASIA) Impairment Scale (AIS) [36]. The AIS of 14% of the participants was unknown, either because it was not detailed [28] or because they were people with pathologies other than SCI [29,30,31]. A high percentage (86%) were AIS A or B (i.e., complete motor injuries), representing 68% of the total subjects included in this review.

All but one of the studies [28] reported the level of injury (LOI) of the participants: 15% had tetraplegia, 28% had high paraplegia (T5 or higher), 49% had low paraplegia (T6 or lower), and the remaining 8% were only specified as having paraplegia.

Three of the studies were conducted on athletes competing nationally and internationally [29,30,31] in different disciplines: wheelchair racing [29,30], handcycling [29,31], and triathlon [31]. All of the studies except that of Jacobs et al. (2021) [34] provided absolute values of VO_2peak_, and all except that of Kressler et al. (2012) [28] also offered values relative to body mass [33,34]. Thus, the mean value of VO_2peak_ was 1.86 L∙min^−1^, although the range was between 0.75 and 2.60 L∙min^−1^, where the lowest value corresponded to subjects with tetraplegia [35].

### 3.3. Exercise Modalities

Two different types of exercise were found depending on the wheelchair exercise protocol used:

Arm cycle ergometer: This can be synchronous (i.e., the two arms of the ergometer are in the same position) or asynchronous (i.e., the arms of the ergometer are placed in opposite positions). Synchronous movement has been shown to be more efficient [37], although the most common system used in the laboratory is asynchronous [29,32,33,37,38,39,40].

Wheelchair propulsion exercise: With a computerized ergometer [41,42], on a treadmill [43] or on rollers [44].

The included studies used arm cycle ergometer exercise with asymmetric movement [28,29,32,33,34,35], wheelchair propulsion on a treadmill [30], and a combination of the two that consisted of handcycle propulsion on a treadmill [31].

### 3.4. Test Protocols

Only five studies [28,32,33,34,35] performed an incremental test similar to the one described by Achten et al. in 2002 [24]. This incremental protocol used the arm cycle ergometer exercise modality, with an initial load of 0 W [28,32,35], 20 W [33], or even 50 W in trained subjects [34]. The duration of each stage was 2 min [28,35] or 3 min [32,33,34], and the load increments were 5 W [35], 10 W [28,35], 15 W [32,33], or 20 W [34]. The other studies [29,30,31] performed tests with stages of 20 min at three intensities: 55%, 65%, and 75% VO_2peak_ [29,30,31]. The modalities of exercise were wheelchair propulsion on a treadmill [30], handcycle on a treadmill [31], and arm cycle ergometer [29].

### 3.5. PFO and Fat_max_

Five of the studies included PFO data calculated from an incremental exercise test [28,32,33,34,35]. SCI participants obtained PFO between 0.06 and 0.30 g∙min^−1^. The lowest rates corresponded to non-trained subjects with cervical SCI (0.06 g∙min^−1^) [35]. The three studies conducted in athletes [29,30,31] determined fat oxidation rates at intensities of 55%, 65%, or 75% VO_2peak_, so PFO could have been out of this range. The paraplegic subjects showed the highest PFO (0.28 g∙min^−1^) [31], while the studies that included tetraplegic subjects showed the lowest values (0.22 vs. 0.28 g∙min^−1^) [29,30].

Only three studies provided Fat_max_ data [32,33,34], obtaining PFO at 41%, 34%, and 51% VO_2peak_, respectively.

## 4. Discussion

There are few studies that have analyzed the oxidation of energy substrates during exercise in people with SCI. In addition, very few offer accurate data about PFO and Fat_max_. In general, they show values below those observed in studies carried out in non-injured populations, although they are significantly better when compared with non-injured subjects who perform the same type of exercise. Nevertheless, due to the heterogeneity of the studies, it was not possible to perform a meta-analysis of the results.

### 4.1. Sex

Sex is a factor that influences the ability to oxidize fat during exercise. It is known that although men obtain higher absolute PFO, women seem to have a higher Fat_max_ (56 vs. 51% VO_2peak_) and a higher PFO relative to lean mass [17]. To the best of our knowledge, there are no studies comparing men and women with SCI; we can find studies carried out either only on men [29,31,32,33] or including both sexes in the same group [28,30,35], but none of the studies included in this review provide separate or comparative data between men and women. In consequence, there is a lack of evidence about how sex affects fat oxidation in SCI people, and about which are the specific normative values for men and women.

### 4.2. Level and Degree of Injury

The level of injury determines which muscle groups are paralyzed and which corporal functions are impaired. Therefore, the ability to perform physical exercise will be different depending on the level of injury. People with cervical SCI present impaired respiratory function [21] and different degrees of paralysis of the upper extremities. Due to the impairment of the sympathetic nervous system in these subjects, venous return, blood pressure, and thermoregulation are affected, and there is also a decrease in the release of catecholamines [45,46]. Therefore, physiological adaptations to exercise will be restricted more significantly in people with tetraplegia than in those with paraplegia or without SCI.

According to the AIS, five degrees of injury exist: A, B, C, D, or E, where A and B are characterized by the absence of voluntary motor activity below the level of injury. Therefore, it is important to describe the degree of injury in studies carried out on SCI populations, due to the metabolic and physiological implications [10,11,47]. In this regard, most studies were conducted on people with complete or severe motor injury [28,32,33,34], but in some cases they included pathologies other than SCI [29,30,31], or non-severe motor injuries [35].

Likewise, higher and complete SCI are associated with greater muscular, respiratory, cardiovascular, and neurovegetative effects. People with tetraplegia will have a lower maximum heart rate and VO_2peak_ than those with paraplegia [48]. According to some studies [9,49], there are significant differences in VO_2peak_ among people with tetraplegia, high paraplegia, and low paraplegia, along with a moderate positive correlation (r = 0.66) between level of injury and VO_2peak_. These same differences in VO_2peak_ were observed in the studies included in this review, with lower values in subjects with cervical SCI. Thus, the limited existing evidence shows that individuals with cervical SCI have an impaired ability to oxidize fat. However, there are no studies comparing cervical versus thoracolumbar SCI, nor complete and incomplete injuries, to determine whether the level and extent of injury affect fat metabolism during exercise.

### 4.3. Physical Condition

Physical condition is an important factor to consider, since it has an essential influence on PFO and Fat_max_ [50]. In the studies carried out with athletes, all of them showed a similar level of training as indicated by their VO_2peak_ values (mean of 2.44 L∙min^−1^), and the highest VO_2peak_ was 2.60 L∙min^−1^ in the only study without tetraplegic participants [31]. It is important to emphasize that during upper-limb exercise, peak performance depends more on local muscle fatigue than on central cardiorespiratory mechanisms [51,52]. Moreover, the muscle mass involved is very small compared to that used during lower-limb exercise. Therefore, the maximum VO_2_ values obtained through upper-limb protocols will be lower than those achieved with more global exercises [53]. Nevertheless, studies comparing individuals with SCI and different levels of fitness could determine specific baseline values for VO_2peak_, PFO, and Fat_max_, which would allow training protocols to be adjusted to optimize fat oxidation and physiological adaptations to exercise.

### 4.4. The Fat_max_ Test

In 2002, Achten et al. developed the Fat_max_ test, which connects PFO and the intensity of exercise achieved [24]. Many authors have modified this protocol, varying the starting load and the magnitude of the increments to adapt it to diverse populations and different levels of training. In addition, it has been described with different kinds of exercise, e.g., cycle ergometer [54], treadmill [55], or arm cycle ergometer [56]. This has made it possible to establish reference values for PFO and Fat_max_ according to the level of training and the type of exercise performed. It would be interesting to compare PFO and Fat_max_ in different modalities to optimize exercise programs for people with SCI. Nevertheless, despite the fact that it has been widely used in the general population [17], this protocol has hardly been used in SCI. The studies that performed incremental tests included a higher range of intensities [28,32,33,34,35], allowing a more accurate calculation of PFO and the Fat_max_ point than those that performed a test at three fixed intensities [29,30,31]. However, the studies at three intensities have also been included in the present review because they are an approximation of an incremental test and provide data on the outcome measures that are focus of this review. Considering that the exercise modality in individuals with SCI is mainly limited to upper-limb exercises, and that their response to exercise is conditioned by the cardiovascular and autonomic nervous system alterations mentioned above, the Fat_max_ test should be adapted in SCI populations to accurately determine PFO and Fat_max_.

### 4.5. PFO

The absolute values of PFO observed in the SCI population of this review are far from those recorded in untrained, non-injured populations performing incremental tests on cycle ergometers (from 0.46 to 0.58 g∙min^−1^ in men; from 0.35 to 0.45 g∙min^−1^ in women) [17]. Similarly, four studies [29,31,32,33] included a control group of non-injured subjects; however, only Jacobs et al. (2013) [32] and Martín-Manjarrés et al. (2021) [33] compared both groups performing the same kind of exercise (i.e., arm cycle ergometer). Jacobs et al. (2013) [32] included a sample of 10 sedentary SCI men and 7 non-injured moderately active but untrained subjects (6 men and 1 woman). The SCI group had a greater ability to oxidize fat (0.13 vs. 0.06 g∙min^−1^) and at higher intensity (41 vs. 13% VO_2peak_) than the control group, despite the significant differences in VO_2peak_ (1.45 vs. 2.10 L/min) and age (45.10 vs. 30.30 years). Later, and following the same approach, Jacobs et al. (2021) [34] showed a PFO of 0.30 g∙min^−1^ and a Fat_max_ of 50.7% VO_2peak_ in subjects with SCI, which is closely matched to the values obtained for athletes; thus, the sample of this study was probably in a good physical condition. Martín-Manjarrés et al. (2021) [33] included 21 SCI men and 20 non-injured men matched by physical condition. They also obtained greater PFO in the SCI group compared to the non-injured group (0.22 vs. 0.17 g∙min^−1^)—both in absolute values and relative to whole-body lean mass and upper-body lean mass. Moreover, these differences persisted even when adjusting for VO_2peak_ and fat mass. Meanwhile, Knechtle et al. carried out three studies on athletes with SCI [29,30,31], although they also included subjects with poliomyelitis, spina bifida, and amputees. In two of these studies, a control group of athletes without SCI was also included. However, both groups did not perform the same type of exercise; whereas athletes with SCI performed an upper-limb exercise on a handcycle [31] or an arm cycle ergometer [29], the athletes without SCI performed an exercise on a cycle ergometer [29,31]. Consequently, there were huge differences in VO_2peak_ (2.35–2.61 vs. 4.45–4.57 L∙min^−1^) and PFO (0.24–0.29 vs. 0.64–0.70 g∙min^−1^) between groups. This can be explained by the differences in the volume of muscle mass involved in the exercise in each case [51], the lower metabolic efficiency, and the limited capacity to extract oxygen from the muscles in the arms compared to those in the legs [57,58]. The studies analyzed in this review show significant variability in PFO, although there are many factors related to the characteristics of the subjects included in the studies that could justify these differences, such as LOI, sex, type of exercise, the inclusion of non-SCI individuals, or the differences in the physical condition of the participants.

### 4.6. Fat_max_

The three studies that provide data regarding Fat_max_ [32,33,34] involved an incremental protocol adapted to an exercise on an arm cycle ergometer. The other studies carried out on athletes [29,30,31] used a protocol that consisted of three 20-minute stages at 55%, 65%, and 75% of VO_2peak_, respectively, which did not offer a sufficient range of intensities to accurately locate the PFO point. In two of these studies, the highest fat oxidation occurred at the 55%VO_2peak_ stage [30,31] compared with the stages at 65% and 75% VO_2peak_; in the other, it was at 75%VO_2peak_ [29]. Nevertheless, lower intensities were not evaluated and, in consequence, it is not possible to confirm that PFO did not occur outside the range of intensities evaluated. Kressler et al. (2014) [35] also carried out an incremental protocol analyzing only the first three stages, since most subjects reached values of respiratory exchange ratio (RER) > 1 in the fourth stage. Similarly, PFO occurred in the first stage which, according to their data, corresponded to 56% VO_2peak_, suggesting that Fat_max_ was around that intensity. The other study by Kressler et al. (2012) [28] again used an incremental protocol with a range of intensity starting from 38% VO_2peak_, which is exactly where the PFO was obtained. This seems to indicate that the Fat_max_ is equal to or less than that value.

In summary, the values of Fat_max_ in the SCI population according to the studies analyzed ranged from 34% to 56% VO_2peak_, although the studies that measured it with more precise protocols obtained values between 34 and 41% VO_2peak_ [32,33]. In the three studies conducted on athletes, the authors did not provide ranges of Fat_max_ values, due to the aforementioned limitations of the protocols used. Therefore, there is limited evidence on Fat_max_ values in SCI populations, since the intensity ranges were not sufficient to determine the PFO.

### 4.7. Limitations

This study has some limitations. The included studies generally had a small and heterogeneous sample (i.e., differences in age, gender, level and type of injury, and physical condition). Moreover, the lack of homogeneity in the protocols used—in terms of both the samples (some studies even included subjects with pathologies other than spinal cord injury) and the outcome measures—made it impossible to perform a meta-analysis. Nevertheless, a strong point of this review is that it provides a vision of the state of the art regarding the oxidation of energy substrates in people with SCI, and it highlights the need for more research focused on the factors that influence the metabolic changes that occur after SCI, as well as how these affect the oxidation of energy substrates during physical exercise.

## 5. Conclusions

Metabolism during exercise has scarcely been studied in individuals with SCI. The existing studies that measure PFO and Fat_max_ in SCI are few, and most of them include small and heterogeneous samples. Similarly, few studies have used adapted Fat_max_ protocols, limiting the ability to accurately understand fat oxidation in these subjects during exercise. Factors such as LOI and degree of injury should be considered when carrying out future studies on the oxidation of energy substrates, along with the physical condition, the differentiation between sexes, and the use of validated and identical protocols between subjects with and without SCI. In general, PFO and Fat_max_ were higher in SCI patients compared to non-injured people when performing the same type of exercise with the upper limbs in both groups (even when fitness levels were lower in those with SCI). Nevertheless, non-injured people performing lower-limb exercise (e.g., cycling, running) showed higher PFO and Fat_max_ than SCI subjects (all highly trained athletes, but still with different fitness levels between groups). In this sense, knowing how the different energy substrates are used during exercise is useful to guide training and to adjust it according to the Fat_max_ values for this population, which would help to maximize the benefits obtained during training—especially with regard to metabolic and cardiovascular problems. More studies—differentiating by sex, age, level and degree of injury, physical condition (including fitness level), lifestyle, associated pathology, and other factors that influence the oxidation of energy substrates—are necessary in SCI patients.

## Figures and Tables

**Table 1:** Quality assessment of the included studies: (**a**) STROBE checklist for observational studies. (**b**). PEDro scale for clinical trials.

(**a**)							
		**Martín-Manjarrés et al., 2021**	**Jacobs et al., 2013**	**Kressler et al., 2012**	**Knechtle et al., 2004a**	**Knechtle et al., 2004b**	**Knechtle et al., 2003**
**1**	**a**	0	1	0	0	0	0
	**b**	1	1	1	1	1	1
**2**		1	1	1	1	1	1
**3**		1	1	1	1	1	1
**4**		1	1	0	1	1	1
**5**		1	1	1	1	1	1
**6**	**a**	1	1	1	1	1	1
	**b**	0	0	0	0	0	0
**7**		1	1	1	1	1	1
**8**		1	1	1	1	1	1
**9**		1	0	0	0	0	0
**10**		0	0	0	0	0	0
**11**		1	1	1	1	1	1
**12**	**a**	1	1	1	1	1	1
	**b**	1	1	1	0	1	1
	**c**	0	0	0	0	0	0
	**d**	0	0	0	0	0	0
	**e**	1	0	0	0	0	0
**13**	**a**	0	0	0	0	0	0
	**b**	0	0	0	0	0	0
	**c**	0	0	0	0	0	0
**14**	**a**	1	1	1	1	1	1
	**b**	1	0	0	0	0	0
	**c**	0	0	0	0	0	0
**15**		1	1	1	1	1	1
**16**	**a**	1	1	1	1	1	1
	**b**	0	0	0	0	0	0
	**c**	0	0	0	0	0	0
**17**		1	1	1	1	1	1
**18**		1	1	1	1	1	1
**19**		1	1	1	0	0	0
**20**		1	1	1	1	1	1
**21**		1	1	1	1	1	1
**22**		1	0	1	0	0	0
**Total**		23	20	19	17	18	18
(**b**)							
		**Jacobs et al., 2021**			**Kressler et al., 2014**		
**1**		1			1		
**2**		0			1		
**3**		1			1		
**4**		1			0		
**5**		0			1		
**6**		0			1		
**7**		0			0		
**8**		1			1		
**9**		1			0		
**10**		1			1		
**11**		1			1		
**Total**		7			8		

**Table 2:** Summary of the included studies.

	Study	Design	Demographic Data	Exercise Modality	Test Protocol	VO_2peak_ (L·min^−1^)	PFO (g·min^−1^)	Fat_max_ (%VO_2peak_)	Conclusions
1	Martín-Manjarrés et al., 2021	Cross-sectional study	N = 41 (21 SCI, 20 non-injured) Sex: 41 men Age *: 32 ± 6 LOI: Th1 to Th12 16 AIS A, 2 AIS B, 3 AIS C	Arm cycle ergometer	Continuous graded exercise: − Initial load: 20 W − Load increments: 15 W every 3 m until RER > 1 − After 5 min rest, increments of 15 W every 1 min until volitional exhaustion (VO_2peak_ determination)	2.16	0.22	34.51%	− People with SCI have a greater ability to oxidize fat during exercise than people without injury with the same level of training − The relative intensity at which PFO occurs is higher in people with SCI than in people without SCI
2	Jacobs et al., 2021	Partially randomized, repeated-measures, crossover design	N = 10 SCI Sex: 10 men Age: 39 ± 10 LOI: Th2 to Th10 7 AIS A, 2 AIS B, 1 AIS C	Arm cycle ergometer	Continuous graded exercise: − Initial load: 0–50 W (depending on the history of each participant) − Load increments: 20 W every 3 min until volitional exhaustion	19.2 mL∙kg^−1^∙min^−1^ (relative to body mass)	0.30	50.7%	− The variability in the data collected is influenced by the large range in age, body mass index, injury duration, and VO_2peak_ of the participants − The assessment of metabolic condition in people with SCI is difficult because exercise is limited in the upper limbs and by the metabolic changes that occur after the injury
3	Kressler et al., 2014	Randomized, double-blinded, placebo-controlled, parallel-group study	N = 11 SCI Sex: 9 men, 2 women Age (2 groups): 37 ± 11; 42 ± 11 LOI: C5 to C8 5 AIS A, 3 AIS C, 3 AIS D	Arm cycle ergometer	Continuous graded exercise: − Initial load: 0 W − Load increments: 5 W (C5–C6) or 10 W (C7–C8) every 2 min until volitional exhaustion or cadence < 50 r.p.m.	0.54	0.065		− Low fat oxidation rate in people with cervical SCI − Resistance training along with intake of protein supplements does not improve fat oxidation
4	Jacobs et al., 2013	Cross-sectional study	N = 17 (10 SCI, 7 non-injured) Sex: 16 men, 1 woman Age: 45 ± 10 LOI: Th4 to Th12 AIS A or B	Arm cycle ergometer	Continuous graded exercise: − 10 min rest. − Initial load: 0 W − Load increments: 15 W every 3 min − Cadence of 55–60 r.p.m.	1.45 17.0 mL∙kg^−1^∙min^−1^ (relative to body mass)	0.13	41%	− The use of the upper limbs in daily activity can improve fat oxidation in subjects with paraplegia − The crossover point occurs earlier compared to the leg cycle ergometer − The modality of exercise influences the ability to oxidize fat more than the condition of paraplegia per se
5	Kressler et al., 2012	Cross-sectional study	N = 12 SCI Sex: 9 men, 3 women Age: 29 ± 7 LOI: Th1 or lower AIS not specified	Arm cycle ergometer	Continuous graded exercise: − 10 min rest − Initial load: 0 W − Load increments: 10 W every 2 min − 5 min recovery	1.29–1.40	0.13	38–41%	− PFO occurs in people with SCI at lower exercise intensities than in non-injured people − The contribution of fats is approximately 50% at very low intensities and suffers a sharp drop (around 15%) at moderate intensities
6	Knechtle et al., 2004 (a)	Cross-sectional study	N = 9 (6 SCI, 2 spina bifida, 1 poliomyelitis) Sex: 7 men, 2 women Age: 38 ± 6 LOI: C5 to Th12 6 AIS A, 1 AIS B	Racing wheelchair propulsion on treadmill	− 3 stages of 20 min − At 55, 65, and 75% VO_2peak_ − 15 min rest after each stage	2.39	0.22	55%	− There are no differences in the rate of fat oxidation between 55% and 75% VO_2peak_ − Fat metabolism training at 55% VO_2peak_ is recommended, since the rate of fat oxidation does not increase at higher intensities
7	Knetchle et al., 2004 (b)	Cross-sectional study	N = 16 (6 SCI, 2 amputees, 8 non-injured) Sex: 8 men Age: 38 ± 5 LOI: Th5 to L1 AIS: 4 A, 2 B	SCI: handcycle on a treadmill Non-injured: cycle ergometer	− 3 stages of 20 min − At 55, 65 and 75% VO_2peak_ − 15 min rest after each stage	2.60	0.28	55%	− VO_2peak_ is higher in cyclists than in athletes with SCI − The contribution of fats to energy expenditure is higher at 55% VO_2peak_ (39%) − PFO is influenced by the musculature involved, the fitness level, the distribution and type of muscle fibers, and the hemodynamic condition
8	Knechtle et al., 2003	Cross-sectional study	N = 20 (8 SCI, 1 spina bifida, 1 amputee, 10 non-injured) Sex: 20 men Age: 42 ± 9 LOI C7 to Th11 AIS: 7 A, 1 B	SCI: handcycle on a treadmill Non-injured: cycle ergometer	− 3 stages of 20 min − At 55, 65, and 75% VO_2peak_ − 15 min rest after each stage	2.35	0.22	75%	− A significant increase in carbohydrate oxidation is observed between 55 and 75% VO_2peak_ − VO_2peak_ is comparable to that of non-injured cyclists, taking into account differences in muscle group size.

* Data referring to SCI subjects are expressed as the mean ± SD; LOI, level of injury; AIS, American Spinal Injury Association (ASIA) Impairment Scale; VO_2peak_, peak oxygen consumption; PFO, peak fat oxidation; RER, respiratory exchange ratio; Fat_max_, %VO_2peak_ at PFO; SCI, spinal cord injury Th, thoracic; C, cervical; r.p.m., revolutions per minute.

## Data Availability

The datasets used and/or analyzed during this study are available from the corresponding author upon reasonable request.
